# Comparison of Chest Radiograph Captions Based on Natural Language Processing vs Completed by Radiologists

**DOI:** 10.1001/jamanetworkopen.2022.55113

**Published:** 2023-02-08

**Authors:** Yaping Zhang, Mingqian Liu, Lu Zhang, Lingyun Wang, Keke Zhao, Shundong Hu, Xu Chen, Xueqian Xie

**Affiliations:** 1 Radiology Department, Shanghai General Hospital, Shanghai Jiao Tong University School of Medicine, Shanghai, China; 2 Winning Health Technology Ltd, Shanghai, China; 3 Radiology Department, Shanghai Sixth People Hospital, Shanghai Jiao Tong University School of Medicine, Shanghai, China

## Abstract

**Importance:**

Artificial intelligence (AI) can interpret abnormal signs in chest radiography (CXR) and generate captions, but a prospective study is needed to examine its practical value.

**Objective:**

To prospectively compare natural language processing (NLP)-generated CXR captions and the diagnostic findings of radiologists.

**Design, Setting, and Participants:**

A multicenter diagnostic study was conducted. The training data set included CXR images and reports retrospectively collected from February 1, 2014, to February 28, 2018. The retrospective test data set included consecutive images and reports from April 1 to July 31, 2019. The prospective test data set included consecutive images and reports from May 1 to September 30, 2021.

**Exposures:**

A bidirectional encoder representation from a transformers model was used to extract language entities and relationships from unstructured CXR reports to establish 23 labels of abnormal signs to train convolutional neural networks. The participants in the prospective test group were randomly assigned to 1 of 3 different caption generation models: a normal template, NLP-generated captions, and rule-based captions based on convolutional neural networks. For each case, a resident drafted the report based on the randomly assigned captions and an experienced radiologist finalized the report blinded to the original captions. A total of 21 residents and 19 radiologists were involved.

**Main Outcomes and Measures:**

Time to write reports based on different caption generation models.

**Results:**

The training data set consisted of 74 082 cases (39 254 [53.0%] women; mean [SD] age, 50.0 [17.1] years). In the retrospective (n = 8126; 4345 [53.5%] women; mean [SD] age, 47.9 [15.9] years) and prospective (n = 5091; 2416 [47.5%] women; mean [SD] age, 45.1 [15.6] years) test data sets, the mean (SD) area under the curve of abnormal signs was 0.87 (0.11) in the retrospective data set and 0.84 (0.09) in the prospective data set. The residents’ mean (SD) reporting time using the NLP-generated model was 283 (37) seconds—significantly shorter than the normal template (347 [58] seconds; *P* < .001) and the rule-based model (296 [46] seconds; *P* < .001). The NLP-generated captions showed the highest similarity to the final reports with a mean (SD) bilingual evaluation understudy score of 0.69 (0.24)—significantly higher than the normal template (0.37 [0.09]; *P* < .001) and the rule-based model (0.57 [0.19]; *P* < .001).

**Conclusions and Relevance:**

In this diagnostic study of NLP-generated CXR captions, prior information provided by NLP was associated with greater efficiency in the reporting process, while maintaining good consistency with the findings of radiologists.

## Key Points

**Question:**

Can natural language processing (NLP) be used to generate chest radiograph (CXR) captions?

**Findings:**

In this diagnostic study including 74 082 CXR cases labeled with NLP for 23 abnormal signs to train convolutional neural networks, an independent prospective test data set of 5091 participants was examined. The reporting time using NLP-generated captions as prior information was 283 seconds, significantly shorter than the normal template (347 seconds) and rule-based model (296 seconds), while maintaining good consistency with radiologists.

**Meaning:**

The findings of this study suggest that NLP can be used to generate CXR captions, which provides a priori information for writing reports and may make CXR interpretation more efficient.

## Teaser

This diagnostic study evaluates chest radiography captions generated by natural language processing compared with the diagnostic findings of radiologists.

## Introduction

Chest radiography (CXR) accounts for 26% of imaging examinations of pulmonary and cardiac diseases. However, the interpretation of CXR findings is challenging because it mainly depends on the expertise of radiologists.^1,2^ The increasing CXR orders and the lack of experienced radiologists, especially in community clinics or primary hospitals, limit the clinical application of CXR.^3^

The development of artificial intelligence (AI) accelerates the automatic interpretation of CXR.^4^ Artificial intelligence solutions based on convolutional neural network (CNN) have shown excellent performance in diagnosing pulmonary diseases,^5,6,7^ identifying the position of feeding tubes,^8^ and predicting the temporal changes of imaging findings.^9^ Studies reported that AI-assisted CXR interpretation improved the diagnostic performance compared with that by a single reader,^10,11^ shortened reporting time,^12^ and helped junior radiologists to write reports.^13^ However, CNN image classification usually relies on supervised training based on expert annotation.^14,15^ Radiology reports contain imaging findings and diagnoses of clinical experts, but these reports are usually unstructured natural text and cannot be directly used for label classification in CNN.

Recently, the bidirectional encoder representations from transformers (BERT) have been developed for natural language processing (NLP),^16^ which greatly improves the ability to recognize semantics and context and can generate medical reports. Fonollà et al^17^ presented an AI-aided system that incorporated a BERT-based image captioning block to automatically describe colorectal polyps in colonoscopy. Xue et al^18^ applied a recurrent generative model to a public data set to generate the imaging description paragraphs and impression sentences of CXR reports. Despite the recent research advances, AI-assisted CXR interpretation has not been routinely used in clinical practice, because this task remains highly challenging.

It is increasingly recognized that AI-involved applications need to undergo a rigorous prospective evaluation to demonstrate their effectiveness. Since most previous studies on CXR interpretation were retrospective tests on selected public data sets,^19,20^ a prospective study in a clinical practice setting is needed to evaluate AI-assisted CXR interpretation. Therefore, we applied the BERT model to extract language entities and associations from unstructured radiology reports to train CNNs and generated free-text descriptive captions using NLP. We randomly assigned a normal template, NLP-generated captions, or rule-based captions to CXR cases in the test group to evaluate the consistency between the generated captions and the final reports of radiologists. The hypothesis is that NLP-generated captions can assist CXR reporting.

## Methods

**Figure 1.**
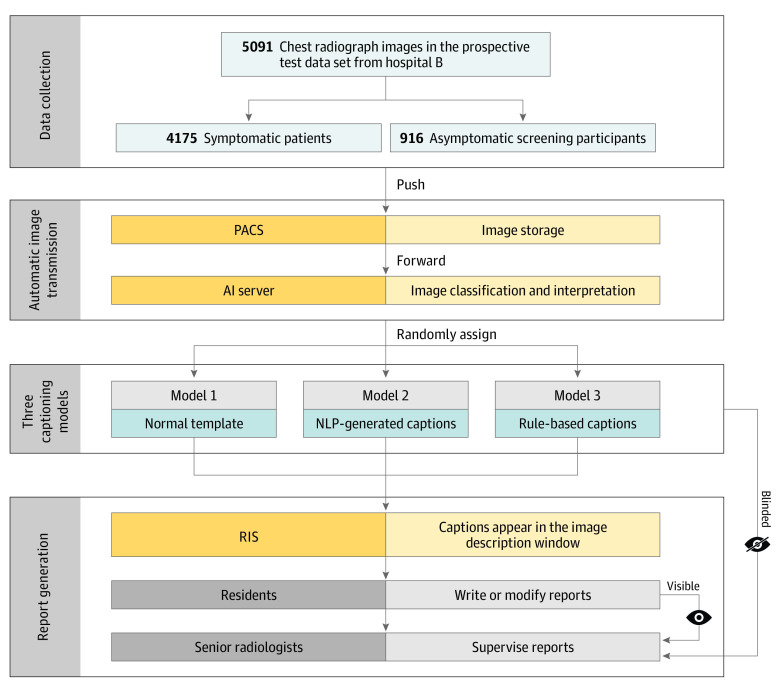
Study Workflow Diagram Colors represent different actors or actions. Dark yellow represents equipment or system; light yellow represents the function of the equipment or system; dark blue represents the captioning models; light blue represents the datasets; dark gray represents the operator; and light gray represents the operator's work. AI indicates artificial intelligence; NLP, natural language processing; PACS, picture archiving and communication system; and RIS, radiology information system.

This study followed the Transparent Reporting of Evaluations With Nonrandomized Designs (TREND) reporting guideline for diagnostic studies. The institutional review board of Shanghai General Hospital, Shanghai Jiao Tong University School of Medicine, approved this study waived the need for informed consent because information prior to the routine reporting process does not pose any risk to the patients. Figure 1 shows the study workflow.

### Retrospective Data Sets

The training data set consisted of consecutive symptomatic CXR cases at hospital A from February 1, 2014, to February 28, 2018. The inclusion criteria were patients (age ≥18 years) with symptoms who underwent posteroanterior CXR for cardiothoracic symptoms, such as chest tightness, cough, fever, and chest pain. The exclusion criteria were mobile CXR, poor image quality, and incomplete reports not drafted and confirmed by 2 radiologists.

The retrospective test data set consisted of CXR cases at hospital B from April 1 to July 31, 2019, including symptomatic patients and asymptomatic screening participants. The symptomatic patients were from emergency, inpatient, and outpatient settings who met the indications for CXR. The asymptomatic participants were from the screening center. The inclusion and exclusion criteria were similar to those of the training data set, except that the screening participants were asymptomatic.

The CXR images were retrieved from the picture archiving and communication system and the corresponding diagnostic reports were retrieved from the radiology information system. eTable 1 in Supplement 1 reports the digital radiography systems used in this study. If a patient had multiple CXR examinations, only the most recent one was included. For each case, a resident drafted a diagnostic report and an experienced radiologist supervised to finalize it. In this way, a total of 67 residents and 20 radiologists participated in reporting at hospital A, and 21 residents and 19 radiologists participated in reporting at hospital B. Due to the actual clinical environment, all involved physicians can view the medical history and previous imaging examinations.

### Prospective Testing

From May 1 to September 30, 2021, the consecutive patients and screening participants in hospital B who underwent CXR were prospectively included. After CXR images were obtained and stored in the picture archiving and communication system, the posteroanterior CXR images were automatically forwarded to the AI server to generate captions. The AI server randomly assigned the images to 1 of the 3 captioning models in a ratio of approximately 1:1:1, including a normal template, NLP-generated captions according to the CNN classification results, and rule-based captions from the CNN results.

When the residents read CXR cases, the caption from 1 of the above 3 models appeared in the imaging-finding window of the radiology information system. The residents can modify the text if necessary. For the normal template, the residents wrote reports based on the template. For the cases with prior CNN results, the residents retained or modified the captions according to their own observations. The start and completion times of image reading by residents were recorded to compare the reporting time based on the 3 models.

After the residents submitted the reports, the senior radiologists observed the CXR images and confirmed the reports. In this process, the senior radiologists were blinded to the AI captioning models, that is, they only viewed the reports written by residents but did not know which model the caption originally came from. Therefore, 19 residents (including L.Z. and L.W.) and 17 radiologists (including Y.Z. and X.X.) participated in reporting.

### BERT-Based CXR Image Labeling

We used the BERT model^21,22^ to recognize language entities, entity span, semantic type of entities, and semantic relationships between language entities. BERT relies on a transformer, an attention mechanism for learning the contextual relationships between words in a text. The BERT model is designed to pretrain the deep bidirectional representation from unstructured text through the joint adjustment of left and right contexts. Therefore, the pretrained BERT model can be fine-tuned through additional output layers to complete the NLP tasks in this study, ie, to learn the semantic information of radiology reports and output semantic recognition vectors for classification.

First, we used BERT to automatically mine all reports in the training data set, segment and extract terms or phrases from the sentences, and cluster them according to semantic distance.^23,24^ Second, 2 radiologists (including Y.Z.) other than the above physicians with 10 and 15 years of experience and 1 NLP engineer (M.L.) reviewed the language clusters to determine whether the terms correctly described the imaging findings on CXR by consensus. They also iteratively ruled out wrong terms and fixed conflicting terms and merged clusters with similar clinical meanings. Finally, a 23-label system of abnormal signs was established, including synonyms, parasynonyms, or phrases that may appear in radiology reports (Box). The details of BERT-based image labeling and CNN algorithm are in the eMethods in Supplement 1.

Box. **Abnormal Signs Extracted by Bidirectional Encoder Representations From Transformers (BERT) Model From Chest Radiograph Reports in the Training Dataset**

**Abnormal sign**

**Lung parenchyma**

Consolidation
Small consolidation
Patchy consolidation
Nodule
Calcification
Mass
Interstitial disease
Cavity
Hilar adenopathy
Emphysema
Pulmonary edema
Thickened bronchovascular markings

**Mediastinum**

Cardiomegaly
Aortic unfolding
Aortic arteriosclerosis

**Pleura**

Pneumothorax
Pleural
  Effusion
  Thickening
  Adhesion
  Calcification

**Thorax**

Scoliosis
Peripherally inserted central catheter implant
Pacemaker implant

### Board Reading

Because most CXR cases lacked pathologic reference and the original CXR reports came from medical staff with various extents of expertise, to establish a solid and unified reference standard to determine the performance of CNN, we reexamined the entire retrospective and prospective test data sets. Two different radiologists (including X.X.) with 21 and 31 years of experience independently reviewed the CXR images and BERT-extracted labels. They made necessary corrections to the labels and resolved the inconsistency by consensus.

### NLP-Based Caption Generation

The caption generation was developed by an NLP-based caption retrieval algorithm. The BERT-based CXR image labeling system generated a 1-hot code for each token sequence in the training data set. In NLP, a token sequence is the grouped characters as a semantic unit for processing.^25^ The token sequences with the same 1-hot code were combined as a subset for caption retrieval. In each subset, the bilingual evaluation understudy (BLEU) score of each token sequence and other token sequences were calculated, and the token sequence with the largest average BLEU score was taken as the caption of this subset. This caption retrieval procedure went through all possible 1-hot combinations in the training data set.

To generate captions in the test data set, the 1-hot code of CNN classification results of each abnormal sign in the CXR image was matched with the subset with the same 1-hot code in the training data set, and the corresponding caption was taken as output. Because the CNN classification model did not provide information about the location and size of abnormal signs, the location descriptions and numbers in the token were left blank.

### Rule-Based Caption Generation

According to the order in which radiologists write reports and the habit of expressing different positive and negative labels, a rule-based caption generation method was proposed (eMethods in Supplement 1). In short, CNN classification results with similar patterns of language description were divided into 8 subcategories to adopt similar expression patterns. For example, subcategory 1 includes the signs of consolidation, small consolidation, patchy consolidation, nodule, calcification, mass, emphysema, pulmonary edema, cavity, and pneumothorax. In this subcategory, each sign with a positive result is directly described. If the CNN determines that a pneumothorax sign is positive, then the rule-based caption is “pneumothorax is observed in the lung.” If all of these signs are negative, the caption is “there are no abnormal densities in both lung fields.” The results of each subcategory are linked as a complete paragraph.

### Similarity Among Captioning Models

The similarity was evaluated using the final report as a reference. Therefore, the BLEU score was calculated to indicate the similarity between the caption (normal template, NLP-generated, or rule-based) and the final report (eMethods in Supplement 1).

### Statistical Analysis

The metrics to indicate the classification performance of CNN included area under the receiver operating characteristic curve (AUC), accuracy, sensitivity, specificity, and F1 score. To supplement the interpretation of the AUC on imbalanced data sets, ie, the high specificity caused by low disease prevalence, we also calculated the area under the precision-recall curve (AUPRC). The 95% CIs were calculated by bootstrapping with 100 iterations to estimate the uncertainty of these metrics.^26^ In this way, the original data were resampled 100 times. Each time, 95% of the data were randomly selected and used to calculate the statistics of interest. Among the 3 groups of patients assigned different caption generation models, the pairwise differences in reporting time and BLEU score were evaluated by independent-sample *t* test. A 2-sided *P* < .05 value was considered statistically significant. MedCalc, version 18 (MedCalc Software) was used for statistical analysis.

## Results

### Study Population

The training data set consisted of 74 082 CXR cases (39 254 [53.0%] women; 34 828 [47.0%] men; mean [SD] age, 50.0 [17.1] years; range 18-102 years) in hospital A (Table). The retrospective test data set consisted of 8126 individuals (3781 [46.5%] men; 4345 [53.5%] women; mean [SD] age, 47.9 [15.9] years; range, 18-92 years) in hospital B, including 5996 (73.8%) symptomatic patients and 2130 (26.2%) asymptomatic screening participants. The prospective test data set included 5091 individuals (2675 [52.5%] men; 2416 [47.5%] women; mean [SD] age, 45.1 [15.6] years; range, 18-98 years) in hospital B, including 4175 (82.0%) symptomatic patients and 916 (18.0%) asymptomatic screening participants.

**Table.** Study Population Characteristics

Variable	No. (%)			Age, mean (SD), y	Positive case, No. (%)
	Total	Men	Women		
Hospital A (training)	74 082	34 828 (47.0)	39 254 (53.0)	50.0 (17.1)	40 743 (55.0)
Hospital B (retrospective testing)					
Symptomatic patients	5996	2964 (49.4)	3032 (50.6)	52.6 (16.7)	2686 (44.8)
Screening participants	2130	817 (38.4)	1313 (61.6)	34.5 (13.6)	206 (9.7)
Hospital B (prospective testing)					
Total symptomatic patients	4175	2250 (53.9)	1925 (46.1)	48.3 (17.0)	1490 (35.7)
Total screening participants	916	425 (46.4)	491 (53.6)	30.5 (9.15)	180 (19.7)
Participants among 3 caption-generating models					
Normal template	1662	858 (51.6)	804 (48.4)	48.9 (17.8)	1081 (65.0)
Symptomatic patients	1367	726 (53.1)	641 (46.9)	52 (16.7)	927 (67.8)
Screening participants	295	137 (46.4)	158 (53.6)	30.6 (9.5)	61 (20.7)
NLP-generated caption	1731	938 (54.2)	793 (45.8)	47.8 (18.2)	987 (57.0)
Symptomatic patients	1413	775 (54.8)	638 (45.2)	51.6 (17.4)	914 (64.7)
Screening participants	318	165 (51.9)	153 (48.1)	30.8 (9.6)	62 (19.5)
Rule-based caption	1698	880 (51.8)	818 (48.2)	48.3 (17.6)	1014 (59.7)
Symptomatic patients	1393	752 (54.0)	641 (46.0)	52.3 (16.6)	934 (67.0)
Screening participants	305	129 (42.3)	176 (57.7)	30.1 (8.3)	57 (18.7)

Abbreviation: NLP, natural language processing.

### Training Data Set

In the training data set, 40 743 of 74 082 (55.0%) cases were abnormal and 33 339 (45.0%) were normal. The abnormal cases included 10 706 (14.5%) with 1 abnormal sign and 30 037 (40.5%) with more than 1 abnormal sign. Among the 23 abnormal signs, thickened bronchovascular markings (37 954 [51.2%]) was the most common, followed by pleural thickening (12 789 [17.3%]), nodule (12 192 [16.5%]), consolidation (9701 [13.1%]), and aortic arteriosclerosis (6837 [9.2%]).

The CNN showed high performance in classifying the 23 abnormal signs. The mean (SD) AUC of these abnormal signs was 0.96 (0.03), ranging from 0.88 (95% CI, 0.87-0.89) to 1.00. The mean (SD) accuracy was 0.95 (0.06); sensitivity, 0.60 (0.25); specificity, 0.96 (0.08); and F1 score, 0.70 (0.20). High AUC values were noted for common abnormal signs, namely, pleural thickening (0.95; 95% CI, 0.94-0.96), nodule (0.88; 95% CI, 0.87-0.89), consolidation (0.94; 95% CI, 0.93-0.95), and aortic arteriosclerosis (0.97; 95% CI, 0.96-0.98).

### Retrospective Test Data Set

In the symptomatic patients (n = 5996) of the retrospective test data set, the mean (SD) AUC of CNN reached 0.87 (0.11); AUPRC, 0.46 (0.15); accuracy, 0.91 (0.08); sensitivity, 0.63 (0.25); specificity, 0.92 (0.08); and F1 score, 0.72 (0.19) (eTable 2 in Supplement 1). The AUCs of major abnormal signs, namely, nodule (0.70; 95% CI, 0.58-0.81), consolidation (0.90; 95% CI, 0.85-0.94), mass (0.98; 95% CI, 0.97-0.99), pneumothorax (0.96; 95% CI, 0.93-0.99), and pleural effusion (0.99; 95% CI, 0.98-0.99), were high.

In the asymptomatic screening participants (n = 2130), the mean (SD) AUC value was 0.89 (0.15); AUPR, 0.38 (0.12); accuracy, 0.93 (0.04); sensitivity, 0.52 (0.26); specificity, 0.98 (0.01); and F1-score, 0.64 (0.25) (eTable 3 in Supplement 1). The AUCs of common abnormal signs, namely, nodule (0.80; 95% CI, 0.72-0.84) and consolidation (0.88; 95% CI, 0.82-0.92), were high.

### Prospective Test Data Set

In the symptomatic patients (n = 4175) of the prospective test data set, 20 abnormal signs were observed (eTable 4 in Supplement 1) and determined by the board reading, in which the most common abnormal signs were peripherally inserted central catheter implant (903 [21.6%]), small consolidation (537 [12.9%]), aortic arteriosclerosis (514 [12.3%]), patchy consolidation (374 [9.0%]), and nodule (235 [5.6%]). In the asymptomatic screening participants (n = 916), 13 abnormal signs were observed, including pleural thickening (140 [15.3%]), scoliosis (13 [1.4%]), pleural effusion (13 [1.4%]), and nodule (9 [1.0%]).

**Figure 2.**
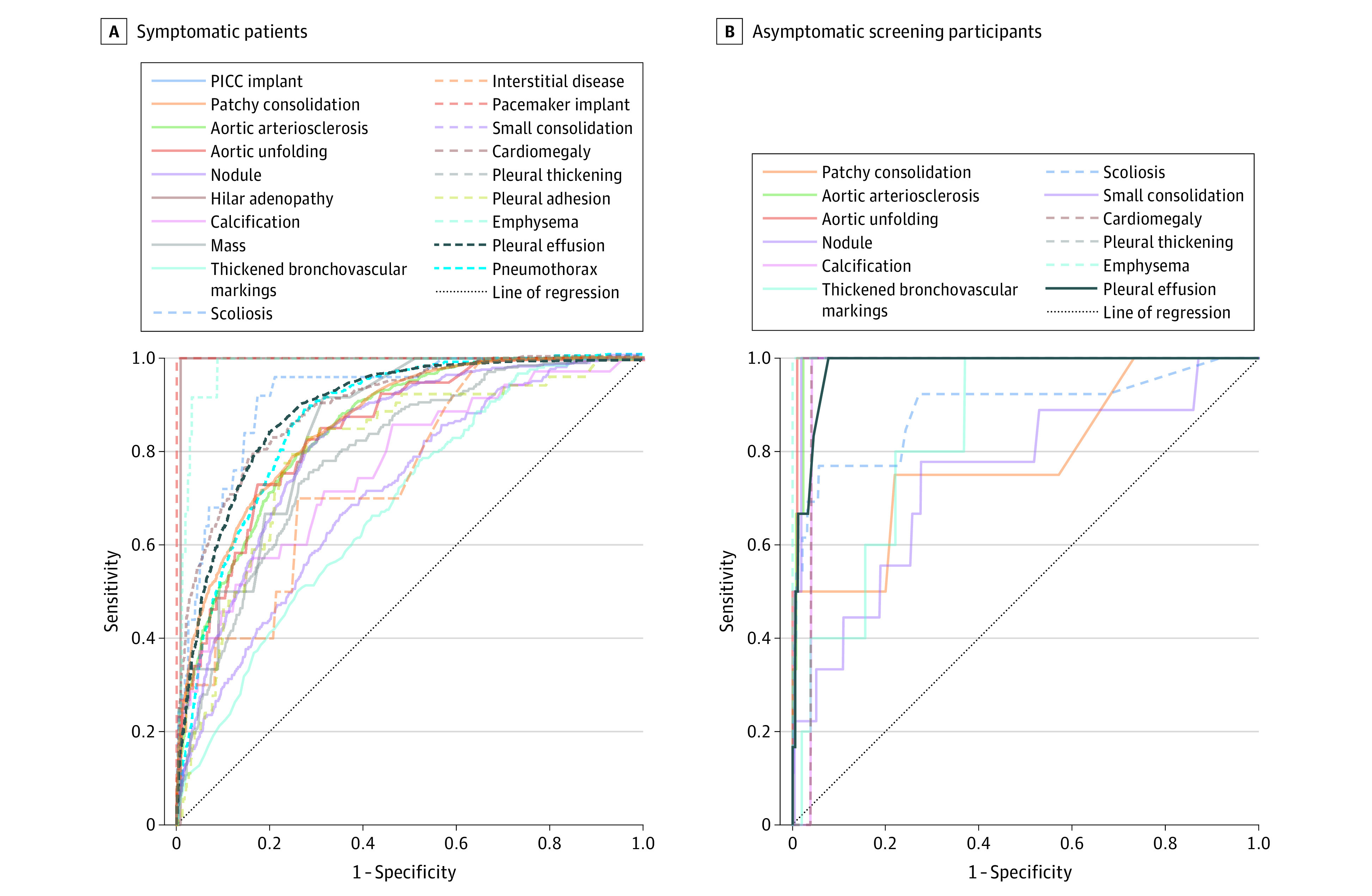
Receiver Operating Characteristic Curves of Convolutional Neural Network Classification in the Prospectively Included Test Data Set Abnormal signs observed in symptomatic patients (A) and screening participants (B). PICC indicates peripherally inserted central catheter.

In the symptomatic patients, the CNN showed high performance in classifying the 20 abnormal signs (Figure 2A; and eTable 5 in Supplement 1), and the mean (SD) AUC of these abnormal signs was 0.84 (0.09), ranging from 0.69 (95% CI, 0.48-0.90) to 0.99 (95% CI, 0.98-1.00). The mean AUPRC was 0.41 (0.19) (eFigure 1A in Supplement 1). The mean accuracy was 0.89 (0.12); sensitivity, 0.47 (0.20); specificity, 0.95 (0.11); and F1 score, 0.60 (0.20).

In the screening participants, the CNN showed high performance in classifying the 13 abnormal signs (Figure 2B; and eTable 6 in Supplement 1), and the mean (SD) AUC was 0.90 (0.13), ranging from 0.52 (95% CI, 0.46-0.58) to 1.00. The mean AUPRC was 0.33 (0.16) (eFigure 1B in Supplement 1). The mean accuracy was 0.96 (0.08); sensitivity, 0.86 (0.20); specificity, 0.96 (0.09); and F1 score, 0.90 (0.15).

### Reporting Time

The residents spent the least reporting time using the NLP-generated captions. The mean (SD) reporting time of residents using the NLP-generated model (283 [37] seconds) was significantly shorter than the normal template (347 [58] seconds; *P* < .001) and rule-based model (296 [46] seconds; *P* < .001). In the normal cases, the reporting time of NLP-generated model (174 [20] seconds) was significantly shorter than the normal template (197 [34] seconds; *P* < .001) but similar to the rule-based model (174 [23] seconds; *P* = .60). In the abnormal cases, the reporting time of the NLP-generated model (456 [71] seconds) was significantly shorter than the normal template (631 [101] seconds; *P* < .001) and rule-based model (531 [97] seconds; *P* < .001).

### Similarity of Captioning Models

**Figure 3.**
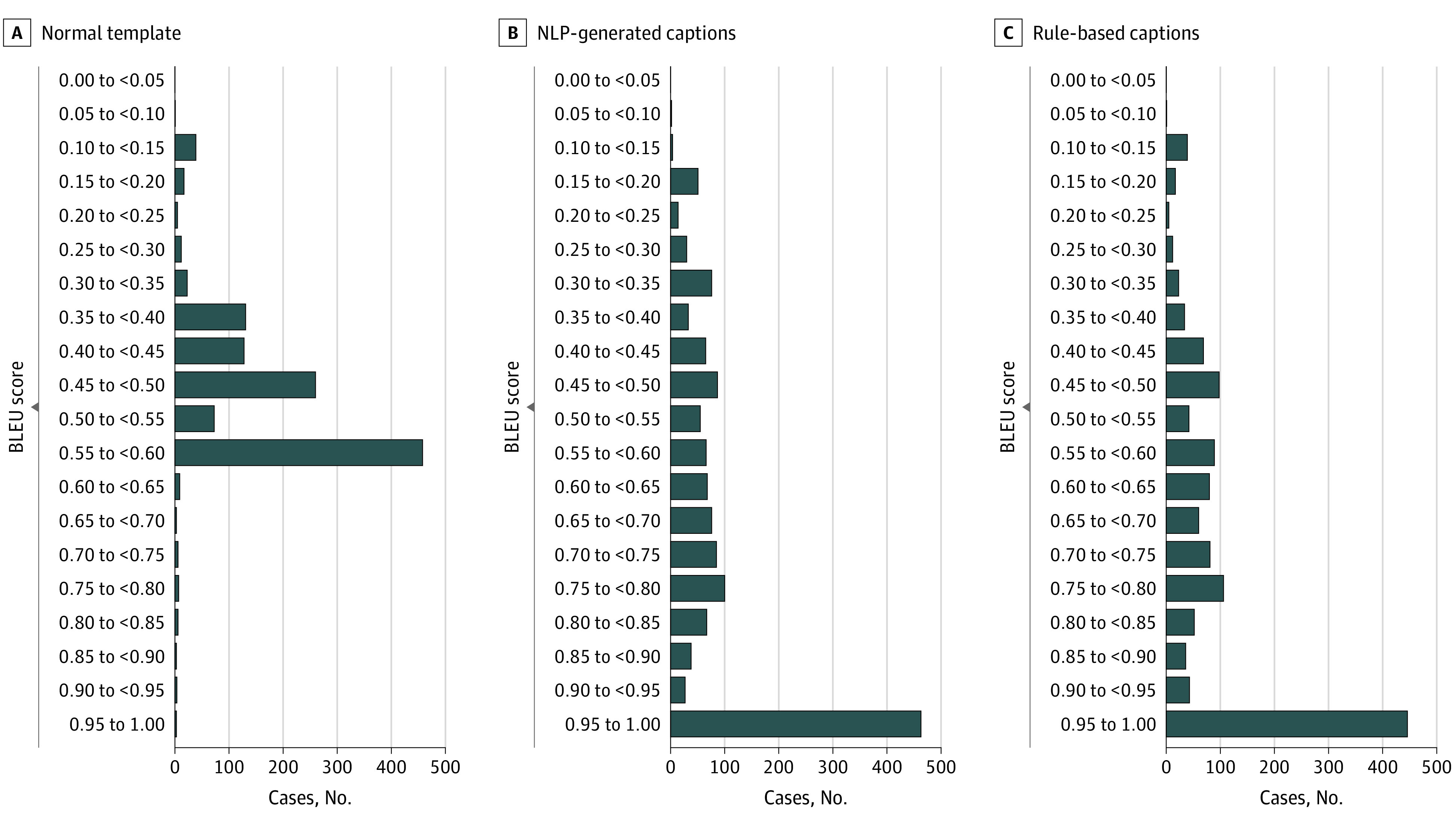
Bilingual Evaluation Understudy (BLEU) Scores in the Prospectively Included Test Data Set Scores shown for normal template (A), natural language processing (NLP)–generated (B), and rule-based (C) captions.

Among the 5091 individuals, the AI server randomly assigned 1662 to a normal template, 1731 to NLP-generated captions, and 1698 to rule-based captions (Figure 3 and the Table). eFigure 2 in Supplement 1 shows some representative cases. The percentage of men and the percentage of abnormal cases (with at least 1 abnormal sign) did not differ significantly among the 3 subgroups (*P* > .05).

The NLP-generated caption was the most similar to the final report, with a mean (SD) BLEU score of 0.69 (0.24), significantly higher than 0.37 (0.09) of the normal template (*P* < .001) and 0.57 (0.19) of the rule-based model (*P* < .001). The BLEU score of the rule-based model was significantly higher than the normal template (*P* < .001) (eTable 7 in Supplement 1).

## Discussion

In this study, we applied the BERT model to extract language entities and relationships from unstructured radiology reports to classify 23 abnormal signs in CXR. In the prospective test data set, the residents spent the least reporting time (283 seconds) using the NLP-generated captions as prior information, which was significantly shorter than the normal template (347 seconds) and rule-based model (296 seconds), especially for the abnormal cases. The NLP-generated captions were the most similar to the final reports, with a BLEU score of 0.69, better than the normal template of 0.37, and the rule-based model of 0.57.

Artificial intelligence has demonstrated its ability in clinical settings on CXR interpretation, including outperforming physicians in detecting major thoracic findings^27,28,29^ and improving the diagnostic sensitivity of residents.^13^ We set up a new implementation scenario, ie, an automatic CXR captioning system that can assist radiologists to write diagnostic reports. The system runs before the traditional reporting workflow and does not alter the care delivery mode. The large number of radiologists involved in the study simulated the actual environment and the heterogeneity of CXR interpretation, which provided a solid foundation for comparing the 3 caption generation models.

The high accuracy of CNN classification is partly attributed to the large number of cases used for model training. The classification model of abnormal signs was trained on more than 70 000 CXR cases. Similarly, some large-scale attempts to train deep-learning models on CXR data also relied on text mining in original radiology reports^4,30^—a process sometimes criticized for the inaccuracy of subjective evaluation.^31^ In our study, 2 experienced radiologists and 1 NLP engineer iteratively refined the language clusters describing the imaging findings on CXR to maximize the accuracy of CXR annotation, while avoiding the huge amount of labor necessary in labeling images from scratch.

Image captioning is the task of describing the content of an image in words.^32^ In this study, we applied NLP-generated image captioning to assist residents to draft diagnostic reports and improve their report efficiency. The mechanism underlying the improved performance of AI-assisted reporting is complex. When multiple abnormal findings are present, the observers are less likely to perceive them all.^33^ Simulation studies have shown that multiclass algorithms can reduce reporting time^34^ and improve the performance of radiology residents in emergency departments.^13^ Therefore, image captions provide residents with a priori information to interpret CXR.

### Strengths and Limitations

The strength of this study is the evaluation of consecutively enrolled individuals in the clinical practice setting. There are some limitations of the study. First, since the residents and radiologists were from the same country, although they are fully qualified specialists, their findings might not be representative of clinicians elsewhere. Although we used the final reports of experienced radiologists as the reference, nonstandard terms in their reports may reduce the BLEU score. Second, we did not include data on ethnicity and patient demographic characteristics beyond age and sex. Future work should be to study the generalizability of this system in different geographic settings. Third, although the current 23 abnormal signs on CXR are common, other abnormal signs need further study to improve the scope and generalizability of the system.

## Conclusions

We developed and integrated an AI-assisted captioning system capable of interpreting multiple abnormal signs on CXR, which provided a priori information for residents and radiologists and was associated with greater efficiency in their work. In this diagnostic study, the NLP-generated CXR captions showed good consistency with expert radiologists, which was better than the commonly used normal template and rule-based model, highlighting the ability of AI-assisted CXR diagnosis. Further research should aim at collecting a broader data set to enhance the quality of the dictionary and the AI models.

## Notes

Supplement 1. **eMethods.** Detailed Methods **eResults.** Detailed Results
eReferences
**eFigure 1.** Precision-Recall Curves of Convolutional Neural Network Classification in the Prospective Test Dataset **eFigure 2.** Three Representative Cases of Different Report Generation Models and Two Cases in Which Errors Occur in the Prospective Test Dataset **eTable 1.** Digital Radiography Systems **eTable 2.** Classification Performance of Convolutional Neural Networks of Symptomatic Patients (n=5,996) in the Retrospective Test Dataset Using Board Reading as the Reference **eTable 3.** Classification Performance of Convolutional Neural Networks of Asymptomatic Screening Participants (n=2,130) in the Retrospective Test Dataset Using Board Reading as the Reference **eTable 4.** Disease Prevalence in the Prospective Test Dataset (n=5,091) Based on Board Reading **eTable 5.** Classification Performance of Convolutional Neural Networks of Symptomatic Patients (n=4,175) in the Prospective Test Dataset Using Board Reading as the Reference **eTable 6.** Classification Performance of Convolutional Neural Networks of Asymptomatic Screening Participants (n=916) in the Prospective Test Using Board Reading as the Reference **eTable 7.** Multiple Regression Analysis on the Significance of Reporting Time and BLEU Score Among Three Models

Supplement 2.
Data Sharing Statement
