# Supplementary material for: Comparison of Chest Radiograph Captions Based on Natural Language Processing vs Completed by Radiologists
Source: JAMA Netw Open. 2023 Feb 8;6(2):e2255113. doi: 10.1001/jamanetworkopen.2022.55113 (PMC9909497; doi:10.1001/jamanetworkopen.2022.55113)
Supplement: Supplement 1. — eMethods. Detailed Methods eResults. Detailed Results eReferences eFigure 1. Precision-Recall Curves of Convolutional Neural Network Classification in the Prospective Test Dataset eFigure 2. Three Representative Cases of Different Report Generation Models and Two Cases in Which Errors Occur in the Prospective Test Dataset eTable 1. Digital Radiography Systems eTable 2. Classification Performance of Convolutional Neural Networks of Symptomatic Patients (n=5,996) in the Retrospective Test Dataset Using Board Reading as the Reference eTable 3. Classification Performance of Convolutional Neural Networks of Asymptomatic Screening Participants (n=2,130) in the Retrospective Test Dataset Using Board Reading as the Reference eTable 4. Disease Prevalence in the Prospective Test Dataset (n=5,091) Based on Board Reading eTable 5. Classification Performance of Convolutional Neural Networks of Symptomatic Patients (n=4,175) in the Prospective Test Dataset Using Board Reading as the Reference eTable 6. Classification Performance of Convolutional Neural Networks of Asymptomatic Screening Participants (n=916) in the Prospective Test Using Board Reading as the Reference eTable 7. Multiple Regression Analysis on the Significance of Reporting Time and BLEU Score Among Three Models [file jamanetwopen-e2255113-s001.pdf]

Supplementary Online Content

Zhang Y, Liu M, Zhang L, et al. Comparison of chest radiograph captions based on natural language processing vs completed by radiologists. *JAMA Netw Open*. 2023;6(2):e2255113. doi:10.1001/jamanetworkopen.2022.55113

eMethods. Detailed Methods

eResults. Detailed Results

eReferences

eFigure 1. Precision-Recall Curves of Convolutional Neural Network Classification in the Prospective Test Dataset

eFigure 2. Three Representative Cases of Different Report Generation Models and Two Cases in Which Errors Occur in the Prospective Test Dataset

eTable 1. Digital Radiography Systems

eTable 2. Classification Performance of Convolutional Neural Networks of Symptomatic Patients (n=5,996) in the Retrospective Test Dataset Using Board Reading as the Reference

eTable 3. Classification Performance of Convolutional Neural Networks of Asymptomatic Screening Participants (n=2,130) in the Retrospective Test Dataset Using Board Reading as the Reference

eTable 4. Disease Prevalence in the Prospective Test Dataset (n=5,091) Based on Board Reading

eTable 5. Classification Performance of Convolutional Neural Networks of Symptomatic Patients (n=4,175) in the Prospective Test Dataset Using Board Reading as the Reference

eTable 6. Classification Performance of Convolutional Neural Networks of Asymptomatic Screening Participants (n=916) in the Prospective Test Using Board Reading as the Reference

eTable 7. Multiple Regression Analysis on the Significance of Reporting Time and BLEU Score Among Three Models

This supplementary material has been provided by the authors to give readers additional information about their work.

© 2023 Zhang Y et al. *JAMA Network Open.*

eMethods. Detailed Methods

Chest radiography equipment

In Hospital-A, five different types of digital radiography systems (Digital Diagnost, Philips; GC85A, Samsung; RADspeed, Shimadzu; CXDI, Canon; and XR220, Optima) were used. In Hospital-B, four different types (XR656, GE; Optima XR220, GE; Platinum 43, DMS; and CXDI, Canon) were used. eTable 1 lists the details of digital radiography systems.

Knowledge graph construction with BERT

We applied the Bidirectional Encoder Representations from Transformers (BERT) model[1] to identify language entities, determine the span of entities, recognize the semantic type of entities, and estimate the relationship among entities. BERT relies on a transformer, which is a kind of attention mechanism to learn the context relationships between words in a text. Through the joint adjustment of contexts, the deep bidirectional representation of unstructured text was used to pre-train the BERT model. Then, the pre-trained BERT model can be fine-tuned to establish corresponding models for various tasks in natural language processing (NLP), such as learning the semantic information of text, and outputting semantic recognition vectors for classification. In our study, language entities refer to the words or phrases that describe anatomical regions (e.g., the lung, mediastinum, and aorta), lesion locations in posteroanterior chest radiography (CXR) (e.g., right, lower, and bilateral), imaging features (e.g., calcification and consolidation), and adjectives of imaging features (e.g., large and patchy). Language relationship refers to the semantic and logical connections among language entities (e.g., right-patchy-consolidation and left-sharp-costophrenic angle).

To ensure the correctness of the language entities and relationships extracted by the BERT model, two experienced radiologists with 31 and 21 years of experience in chest imaging inspected and amended the

© 2023 Zhang Y et al. *JAMA Network Open.*

extracted language entities according to a traditional radiology textbook entitled Radiology and Imaging, 6th edition, and the Fleischner Society's glossary.[2] They also put non-uniform terms into appropriate linguistic entities, such as abbreviations and colloquial terms. The language entities of abnormal signs on CXR were divided into four categories according to anatomical regions, including lung parenchyma, pleura, mediastinum, and chest wall. eFigure 1 shows the representative language entities and relationships in unstructured radiology reports.

The core of knowledge graph is the knowledge model: the collection of interrelated descriptions of concepts, entities, and relationships. A knowledge graph puts data in context through links and semantic metadata, which provides a framework for data integration, analysis, and sharing.[3] In this study, to build a knowledge graph to annotate CXR, language entities were first extracted, and then the relationships between candidate entity pairs were analyzed. We defined some rules to restrict the potential relationships of CXR captioning text. For example, the direct semantic relationship between the name of an anatomical region and an imaging feature was acceptable, but there was no correlation between the adjective of an anatomical region and an imaging feature. Then the boundary relationship between two language entities was weighted to indicate whether this relationship was positive or negative. The extracted terms or phrases were classified as positive (e.g., consolidation in the inferior lobe of the right lung), or positive with uncertainty (e.g., possible or not excluded), and negative (e.g., no abnormal sign). The tuples in the established knowledge graph imply rich semantic relationships between different entity pairs. For example, a semantic and relational tuple can indicate whether there is a nodule in the right lower lobe of the lung.

This study used the natural language reports of different doctors to construct image labels. Although their language styles were variable, their language in radiology reports had a hierarchical relationship when describing chest disorders. To understand this hierarchical relationship, we constructed "anatomical location -

disorder” relationship to help identify entities and extract entity relationships. For example, for the hierarchical relationship between "pleural thickening" and "pleural abnormality", thickening is a subclass of abnormality, thus "pleural thickening" is subordinate to "pleural abnormality".

Building a knowledge graph helps to understand the relationships between different entities, establish labels at different language levels, and merge a large number of synonyms in free-text reports to build language clusters. In addition, we counted the number and frequency of various entities, so as to merge and reclassify the entities with a lower frequency to a higher semantic level. For example, if there were descriptions of “aortic arch calcification” and “aortic wall calcification” in the training dataset, the semantic levels of these two descriptions were lower than their upper-level label “aortic arteriosclerosis”. Therefore, we can merge “aortic arch calcification” and “aortic wall calcification” into the label "aortic arteriosclerosis" for the weakly supervised training of convolutional neural network (CNN) model.

Finally, with the help of the knowledge graph, we configured complex query items according to the semantic relationships to search free-text reports. Keywords, terms, or other manually designed semantic settings make it more accurate to label CXR images from unstructured free-text reports.

Labeling the training dataset

Based on the language clustering extracted from radiology reports by the BERT model, disorder labels were established for CXR images in the training dataset. From the perspective of anatomical regions, the disorder labels included four categories, i.e., lung parenchyma, pleura, mediastinum, and chest wall. The procedure to establish the labels is as follows,

In the first step, all radiology reports in the training dataset were mined using the BERT model to extract all terms with close entity span and construct language clusters. The image descriptions and diagnostic

© 2023 Zhang Y et al. *JAMA Network Open.*

conclusions in radiology reports were combined and divided into multiple sentences. The BERT model automatically extracted terms or phrases from these sentences, and clustered them based on semantic distance. This process produced more than 40 language clusters consisting of synonyms or parasynonyms of abnormal signs or disorders, which described the meaningful and frequently used abnormal signs.

In the second step, the two experienced radiologists mentioned earlier and one experienced NLP engineer held a consensus meeting to review the language clusters to determine whether the terms in them correctly described the imaging findings on CXR. They decided whether a term or phrase belonged to a language cluster based on clinical relevance and dependence. They also iteratively excluded erroneous and conflicting terms from the cluster, and merged clusters with similar clinical meaning. In this way, the language clusters and their subordinate terms or phrases were updated. This process was iterated multiple times until all extracted terms or phrases were correctly categorized and hierarchically associated. During the above iteration process, if a language cluster contained ≥ 50 subjects in the training dataset (except for cavity, which is rare but clinically important), then the language cluster and its affiliated terms or phrases were considered a disorder label to annotate the image for training CNN. The reason for setting the language cluster to ≥ 50 subjects is that the number of positive cases for training CNN should not be too small or imbalanced.

Finally, a labeling system consisting of 23 abnormal signs was established (Figure 2), which contained synonyms or parasynonyms, or phrases that may appear in free-text radiology reports. Then, the 23-label system was used to mark CXR images, and to train and test CNN models.

Labeling the test datasets

Since the free-text reports in the test datasets were unstructured and cannot be directly used as the reference for CNN classification results, we used the BERT model to extract the description of abnormal signs from the

free-text reports. Based on the knowledge graph derived from the training dataset, the image labels of abnormal signs were extracted from the free-text reports of the test datasets. Two radiologists with 21 and 10 years of experience in chest imaging examined and revised the BERT-extracted labels to ensure their consistency with the radiology reports and resolved the discrepancy by consensus.

CNN algorithm

The CNN model used in this study is based on the inception-v4 architecture that was pre-trained on the public ImageNet dataset.[4] The original 1,000 classes in the last fully-connected layer of inception-v4 architecture were replaced by 23 classes, representing the 23 labels we have established previously. Because of the uneven distribution of positive and negative cases under some labels in the training dataset, a customized weighted binary cross-entropy loss function was used to increase the weight of the input data of the minority.

We applied 5-fold stratified cross-validation. Cross-validation provides more information than one training-validation fitting model. Stratified cross-validation allocates the data into splits in a way such that the distribution of the outcome variable is the same across all the different splits.[5] The training in this study was divided into five deep learning processes. In each process, the entire training dataset was divided into 80% for training and 20% for internal validation. Each CNN model was trained for 24 epochs. The mean predictive probability of the 5 inception-v4 models was taken as the final output value. This mean value was then used to determine the threshold between positive and negative results by obtaining the maximum F1 score for each label. This threshold was then used to calculate sensitivity and specificity.

NLP-based caption generation

The caption generation was developed by an NLP-based caption retrieval algorithm. The BERT-based CXR image labeling system generated a one-hot code for each token sequence in the training dataset. In NLP,

© 2023 Zhang Y et al. *JAMA Network Open.*

tokenization is the process of tokenizing or splitting a string, text into a list of tokens. A token is a string of contiguous characters between two spaces, or between a space and punctuation marks.[6] In short, a token sequence is the grouped characters as a semantic unit for processing. The token sequences with the same one-hot code were combined into a subset for caption retrieval. In each subset, the bilingual evaluation understudy (BLEU) score of each token sequence and other token sequences were calculated, and the token sequence with the largest average BLEU score was taken as the caption of this subset. This caption retrieval procedure went through all possible one-hot combinations in the training dataset.

To generate captions in the test dataset, the one-hot code of CNN classification results of each abnormal sign in the CXR image was matched with the subset with the same one-hot code in the training dataset, and the corresponding caption was taken as output. As the CNN classification model didn't provide information about the location and size of abnormal signs, the location descriptions and numbers in the token were left blank.

Rule-based caption generation

A rule-based caption generation algorithm was developed. According to the order in which radiologists write reports and the habit of expressing different positive and negative labels, the algorithm generates captions based on the CNN's positive or negative judgment of an abnormal sign. CNN classification results with similar natural language description patterns are divided into subcategories, as shown below,

Subcategory 1, consolidation, small consolidation, patchy consolidation, nodule, calcification, mass, emphysema, pulmonary edema, cavity, and pneumothorax. In this subcategory, each positive label is directly described. The concatenating procedure follows the listed sequence. For example, if the CNN determines that a "pneumothorax" sign is positive, then the rule-based caption is "Pneumothorax is observed in the lung". If all of these signs are negative, the caption is "There are no abnormal densities in both lung fields."

Subcategory 2, interstitial disease. The caption of a positive “interstitial disease” sign is “Fibrosis is observed in bilateral lung fields.” If the sign is negative, no description is added.

Subcategory 3, scoliosis. The description of a positive “scoliosis” sign is “The thorax is symmetrical. The trachea and mediastinum are in the middle.” The caption of a negative sign is “Scoliosis of the spine is observed, and the trachea and mediastinum are in the middle.”

Subcategory 4, hilar adenopathy. The caption of a positive “hilar adenopathy” sign is “Right/left/bilateral hilar adenopathy is observed.” The caption of a negative sign is “The bilateral hilar structures are normal.”

Subcategory 5, thickened bronchovascular markings. The caption of a positive “thickened bronchovascular markings” sign is “Right/left/bilateral bronchovascular markings are thickened.” The caption of a negative sign is “The bronchovascular markings of both lungs are clear.”

Subcategory 6, aortic unfolding, aortic arteriosclerosis, and cardiomegaly. In this subcategory, each positive label is directly described. The caption of a positive “cardiomegaly” sign is “The heart shadow is enlarged, and the cardiothoracic ratio is [ ].” If all these signs are negative, the caption is “No abnormality has been found in the cardiac shadow and the large mediastinal vessels.”

Subcategory 7, pleural thickening, pleural adhesion, pleural calcification, and pleural effusion. In this subcategory, each positive label is directly described. If all of these signs are negative, the caption is “The bilateral diaphragms are smooth, and bilateral costophrenic angles are sharp.”

Subcategory 8, peripherally inserted central catheter (PICC) implant and pacemaker implant. In this subcategory, each positive label is directly described. If the signs are negative, no description is added.

© 2023 Zhang Y et al. *JAMA Network Open.*

Bilingual evaluation understudy (BLEU)

BLEU has been designed to evaluate the quality of machine translation or the similarity of sentences.[7] In BLEU, uni-gram belongs to the word level, which focuses on the adequacy of translation, that is, to measure the similarity word by word. N-gram is at the level of phrases and sentences, which focuses on the fluency of translation. When phrases and sentences are accurate, the language is naturally relatively fluent. Therefore, we summed and averaged the uni-gram and n-gram to represent the similarity between the model-generated caption and the final report.

The results are evaluated by summing and averaging uni-gram and n-gram

$$\mathrm{BP} = \begin{cases} 1 & \text{if } c > r \\ e^{(1-r/c)} & \text{if } c \le r \end{cases}$$

in which, BP is the brevity penalty value, r is the number of words in the reference sentence, and c is the number of words in the candidate sentence.

$$\mathrm{BLEU} = \mathrm{BP} \times \exp\left(\sum_{n=1}^{N} w_n \log p_n\right)$$

in which, p_n is the geometric average of the modified n-gram precision, and w_n is the positive weights summing to one.

System environment

The code of this study is publicly available at https://zenodo.org/record/5335914#.YUS3pcj1dRG/. We used an open-source tool for deep learning (PyTorch, http://pytorch.org/), and used a computer vision library (OpenCV, http://opencv.org/) and a data analysis library (Scikit-learn, http://scikit-learn.org/). The code runs on Linux platform (Ubuntu 16.04, Canonical Ltd.) with four graphics processing units (GTX 1080Ti, Nvidia) in

© 2023 Zhang Y et al. *JAMA Network Open.*

parallel with a total of 44 GB of graphical memory.

Statistics

In addtion to the statistical analysis in the maintext, we conducted multiple regression analysis on the significance of reporting time and BLEU score among three caption generation models, adjusted for age, gender, referral source, and number of abnormal signs. A statistical software package (MedCalc v18, MedCalc Software) was used for statistical analysis.

© 2023 Zhang Y et al. *JAMA Network Open.*

eResults. Detailed Results

In eTable 7, after adjusting for age (p=0.033), sex (0.014), referral source (p<0.001), and number of abnormal signs (p=0.048), the reporting time of symptomatic patients was significantly associated with caption generation models (p=0.013). After Adjusting for age (p=0.300) and sex (0.083), and number of abnormal signs (p<0.001), the reporting time of asymptomatic screening participants was also significantly associated with caption generation models (p=0.007). After adjusting for age (p=0.923), sex (0.083), referral source (p=0.027), and number of abnormal signs (p<0.001), the BLEU score of symptomatic patients was significantly associated with caption generation models (p=0.034). After adjusting for age (p=0.243), sex (0.653), and number of abnormal signs (p<0.001), the BLEU score of asymptomatic participants was significantly associated with caption generation models (p=0.005). In addition, the multiple regression analysis showed the more abnormal signs, the longer the reporting time, the lower BLEU score.

© 2023 Zhang Y et al. *JAMA Network Open.*

eReferences

1. Rasmy L, Xiang Y, Xie Z, Tao C, Zhi D. Med-BERT: pretrained contextualized embeddings on large-scale structured electronic health records for disease prediction. *NPJ Digit Med*. 2021;4(1):86. doi:10.1038/s41746-021-00455-y
2. Hansell DM, Bankier AA, MacMahon H, McLoud TC, Müller NL, Remy J. Fleischner Society: glossary of terms for thoracic imaging. *Radiology*. 2008;246(3):697-722. doi:10.1148/radiol.2462070712
3. Fei H, Ren Y, Zhang Y, Ji D, Liang X. Enriching contextualized language model from knowledge graph for biomedical information extraction. *Brief Bioinform*. 2021;22(3):bbaa110. doi:10.1093/bib/bbaa110
4. Szegedy C, Ioffe S, Vanhoucke V, Alemi AA. Inception-v4, Inception-ResNet and the Impact of Residual Connections on Learning. *Proceedings of the Thirty-First AAAI Conference on Artificial Intelligence*. 2017:4278-4284.
5. Bey R, Goussault R, Grolleau F, Benchoufi M, Porcher R. Fold-stratified cross-validation for unbiased and privacy-preserving federated learning. *J Am Med Inform Assoc*. 2020;27(8):1244-1251. doi:10.1093/jamia/ocaa096
6. Liu Z, Chen Y, Tang B, et al. Automatic de-identification of electronic medical records using token-level and character-level conditional random fields. *J Biomed Inform*. 2015;58 Suppl(Suppl):S47-S52. doi:10.1016/j.jbi.2015.06.009
7. Ke X, Hu P, Yang C, Zhang R. Human-Machine Multi-Turn Language Dialogue Interaction Based on Deep Learning. *Micromachines (Basel)*. 2022;13(3):355. doi:10.3390/mi13030355

© 2023 Zhang Y et al. *JAMA Network Open.*

eFigure 1. Precision-recall curves of convolutional neural network classification in the prospective test dataset

A. Symptomatic patients

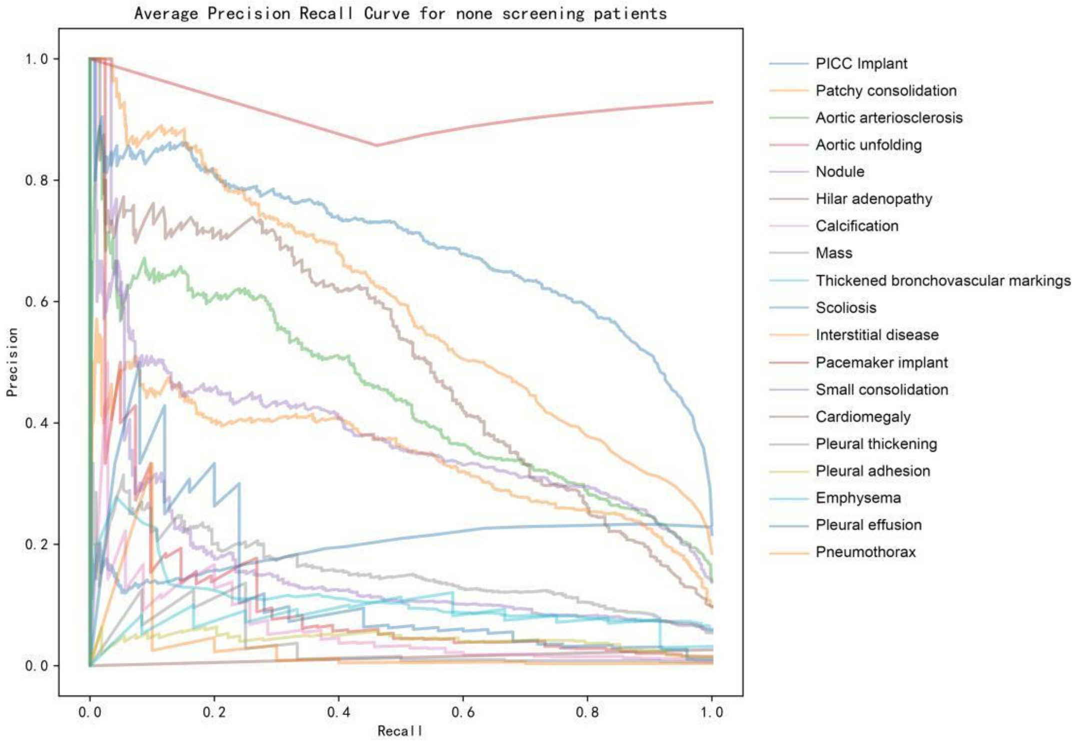

B. Asymptomatic screening participants

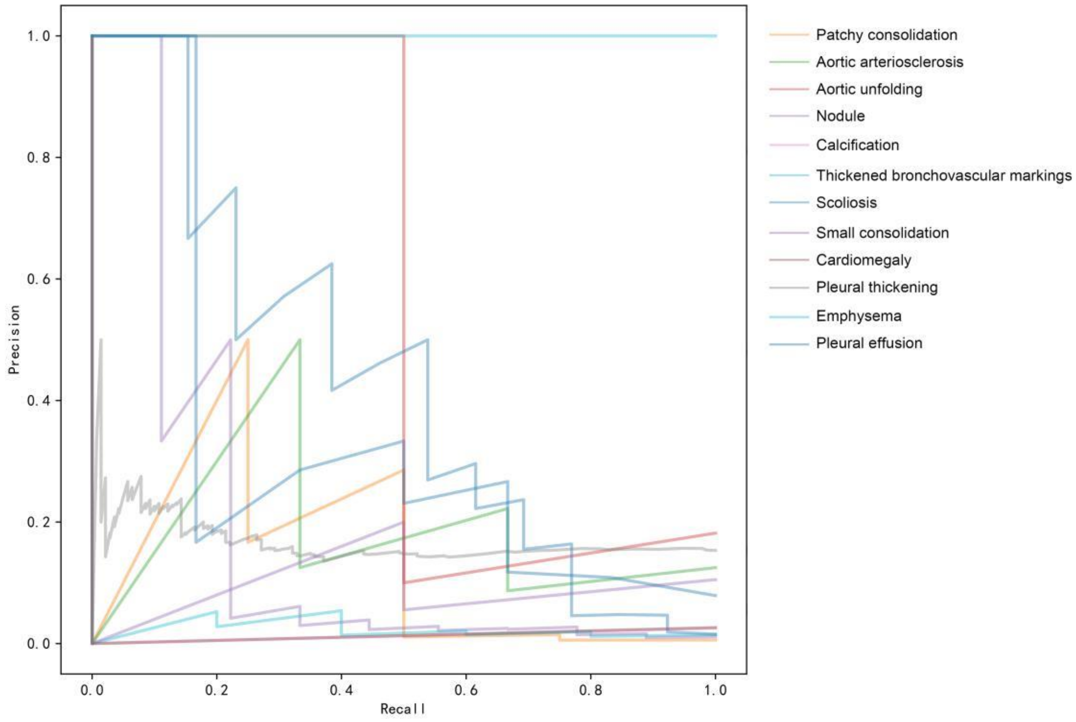

© 2023 Zhang Y et al. *JAMA Network Open.*

eFigure 2. Three representative cases of different report generation models and two cases in which errors occur in the prospective test dataset

A) Male, 35y, who was randomly assigned a normal template of chest radiography, as follows,

"The thorax is symmetrical. The trachea and mediastinum are in the middle. The bronchovascular markings of both lungs are clear. There are no abnormal densities in both lung fields. The bilateral hilar structures are normal. No abnormality has been found in the cardiac shadow and the large mediastinal vessels. The bilateral diaphragms are smooth, and bilateral costophrenic angles are sharp."

The findings in the final diagnostic report are, "The thorax is symmetrical. The trachea and mediastinum are in the middle. Pleural effusion is observed in the right lower lung field and the right lower lung is compressed. The bronchovascular markings of the left lung are clear. The left hilar structures are normal. The cardiac shadow and the large mediastinal vessels are normal. No abnormality is found in the left pleura. The left diaphragm is smooth, and the left costophrenic angle is sharp."

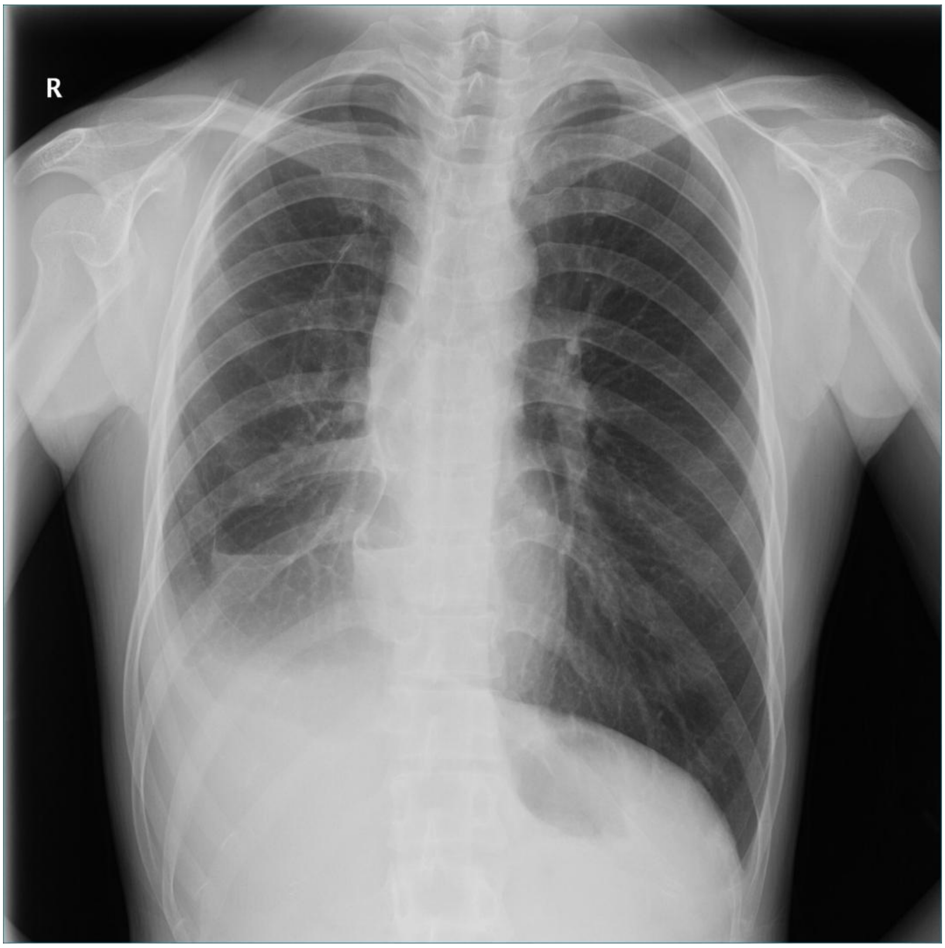

© 2023 Zhang Y et al. *JAMA Network Open.*

B) Female, 79y, who was randomly assigned a natural language processing (NLP)-generated caption, as follows,

"The thorax is symmetrical. The trachea and mediastinum are in the middle. The bilateral hilar structures are normal. The bronchovascular markings of both lungs are enhanced. Patchy shadows are seen in the lung field, with a blurred margin. A small nodule is seen in the lung field. No abnormality has been found in the cardiac shadow and the large mediastinal vessels. The bilateral diaphragms are smooth, and bilateral costophrenic angles are sharp."

The findings in the final diagnostic report are, "The thorax is symmetrical. The trachea and mediastinum are in the middle. The bilateral hilar structures are normal. The bronchovascular markings of both lower lungs are enhanced. Patchy shadows are seen in the upper field of the left lung with a blurred margin. A small nodule is seen in the lung field, sized 11×7mm. No abnormality has been found in the cardiac shadow and the large mediastinal vessels. The bilateral diaphragms are smooth, and bilateral costophrenic angles are sharp."

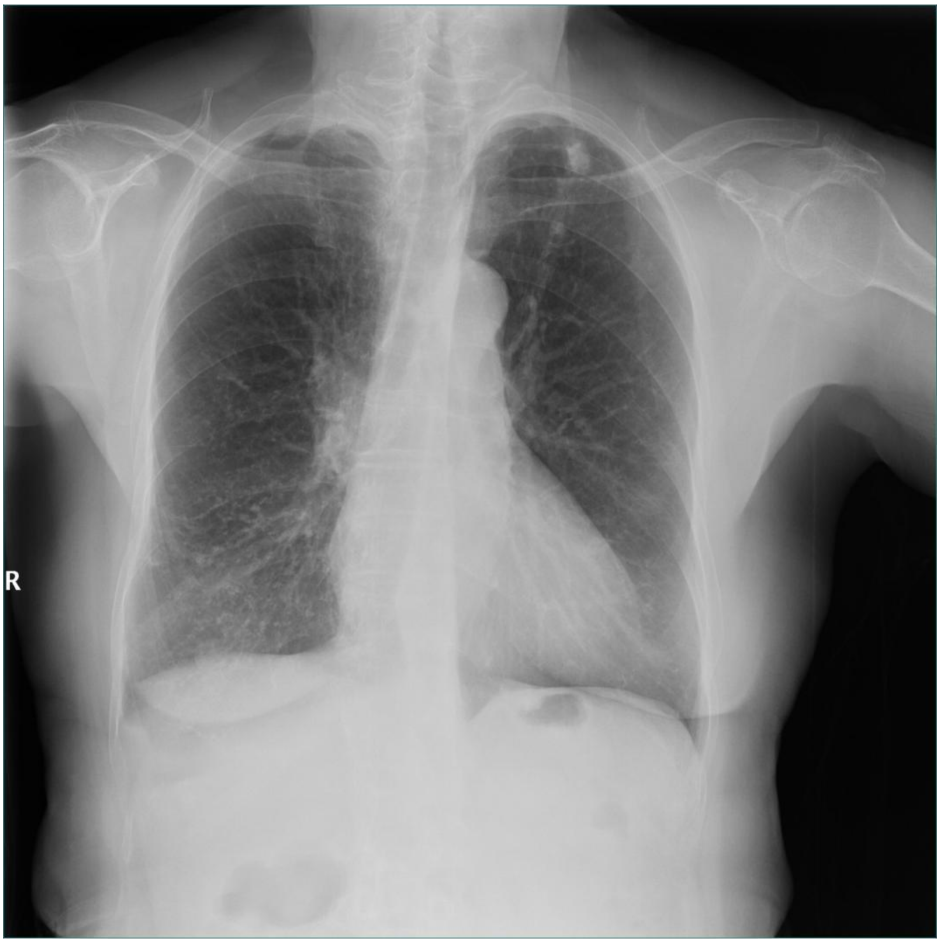

© 2023 Zhang Y et al. *JAMA Network Open.*

C) Female, 80y, who was randomly assigned a rule-based caption, as follows,

“The thorax is symmetrical. The trachea and mediastinum are in the middle. The bronchovascular markings of both lungs are clear. The bilateral hilar structures are normal. The interstitial disease is seen in the lung field. Patchy consolidation is seen in the lung field. A small nodule is seen in the lung field. Aortic arteriosclerosis is seen. No abnormality has been found in the cardiac shadow. Pleural thickening is seen. Pleural adhesion is seen. The bilateral diaphragms are smooth.”

The findings in the final diagnostic report are, “The thorax is symmetrical. The trachea and mediastinum are in the middle. The bronchovascular markings of both lungs are clear. The bilateral hilar structures are normal. Scattered fibrosis lesions are seen in the bilateral lung fields. Patchy consolidation is seen in the upper field of the right lung. A small nodule is seen in the middle field of the right lung. Calcification is seen in the aortic knob. No abnormality has been found in the cardiac shadow. Pleural thickening and adhesion are seen in the left costophrenic angle. The bilateral diaphragms are smooth.”

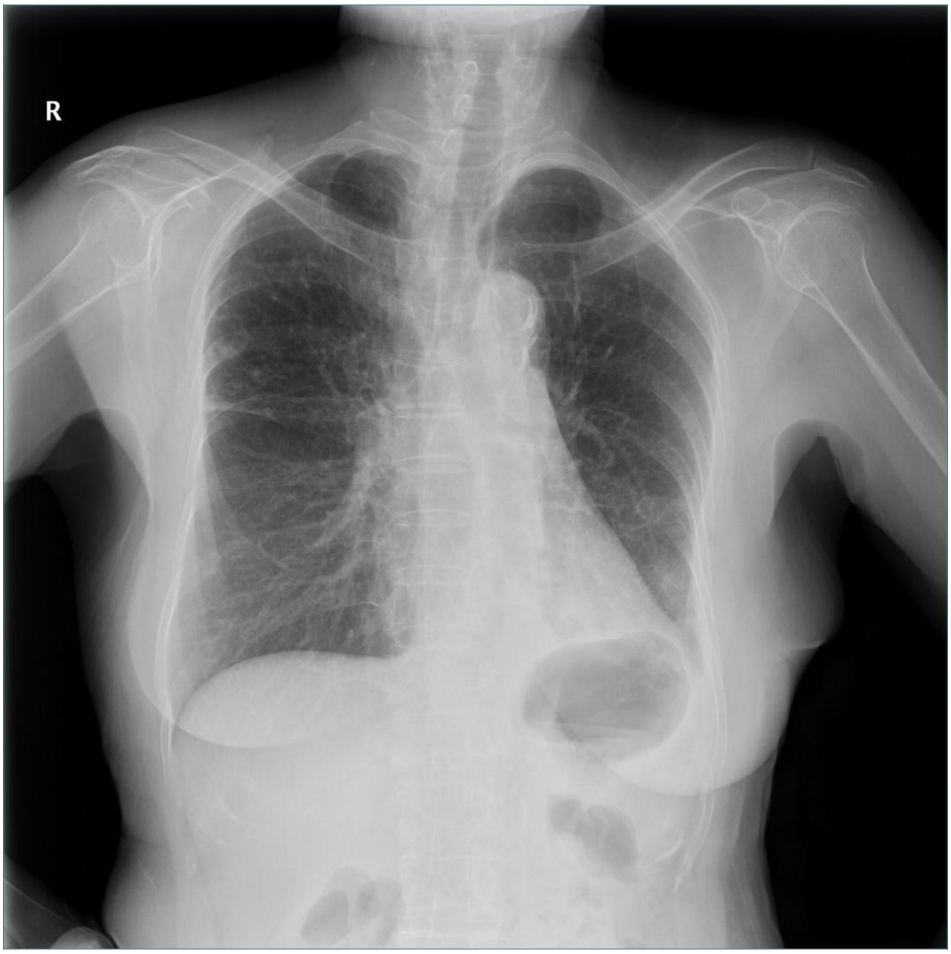

© 2023 Zhang Y et al. *JAMA Network Open.*

D) Female, 74y, who was randomly assigned a NLP-generated caption, as follows,

"The thorax is symmetrical. The trachea and mediastinum are in the middle. The bilateral hilar structures are normal. The bronchovascular markings of both lungs are enhanced. A small nodule is seen in the lung field. No abnormality has been found in the cardiac shadow and the large mediastinal vessels. The bilateral diaphragms are smooth, and bilateral costophrenic angles are sharp."

After reviewing this CXR image, the radiologist considered it a normal case. The nipple was incorrectly recognized as a lung nodule.

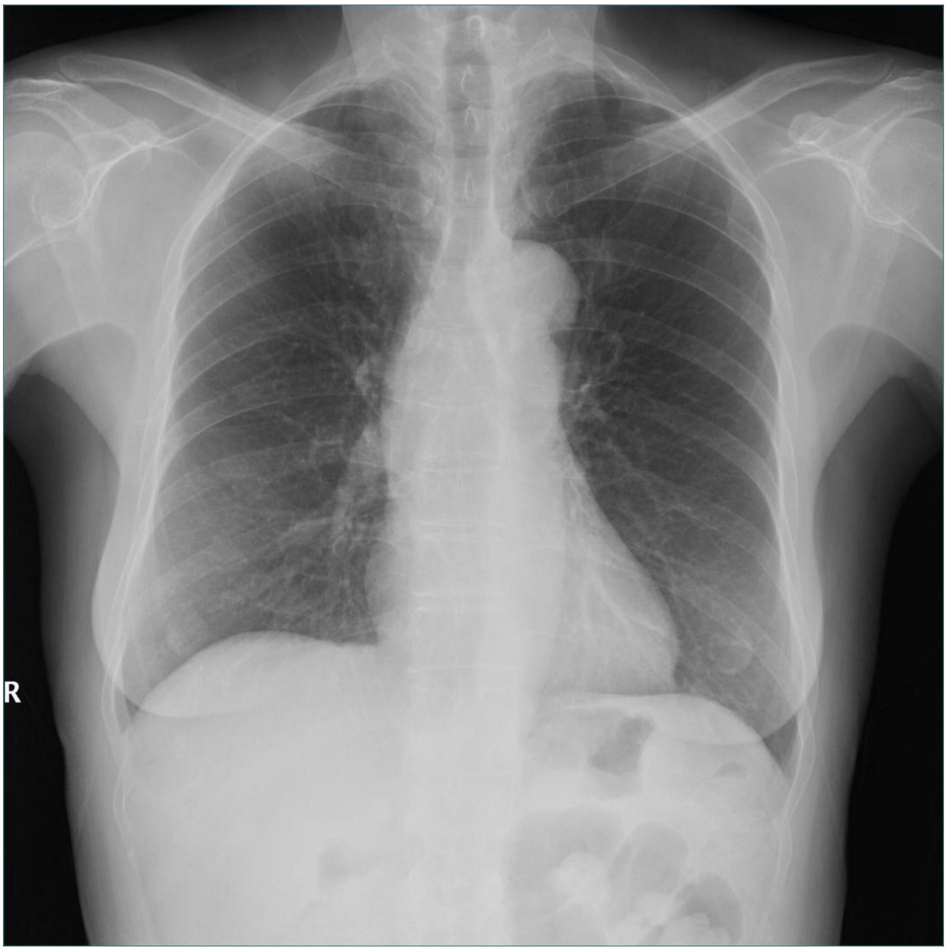

© 2023 Zhang Y et al. *JAMA Network Open.*

E) Male, 62 y, who was randomly assigned a NLP-generated caption, as follows,

"The thorax is symmetrical. The trachea and mediastinum are in the middle. The bronchovascular markings of both lungs are clear. There are no abnormal densities in both lung fields. The bilateral hilar structures are normal. No abnormality has been found in the cardiac shadow and the large mediastinal vessels. The bilateral diaphragms are smooth, and bilateral costophrenic angles are sharp. PICC implant is observed."

After reviewing this CXR image, the radiologist found pleural adhesion in the left costophrenic angle. This patient underwent a CT scan several days later, which confirmed pleural adhesion.

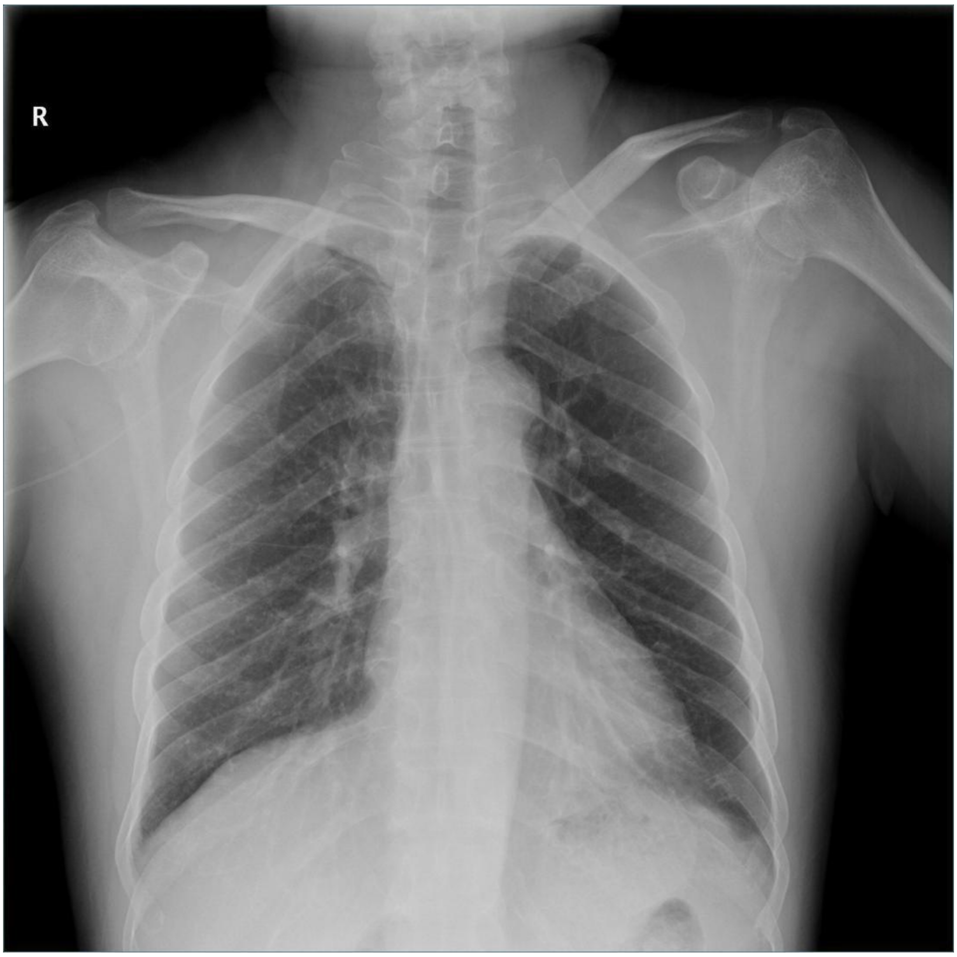

© 2023 Zhang Y et al. *JAMA Network Open.*

eTable 1. Digital radiography systems

Institution	Model of digital radiography	Tube voltage, kVp	Image matrix
Hospital-A	Philips DigitalDiagnost	74	2048×2048
	Samsung GC85A	Unknown	2048×2048
	Shimadzu RADspeed	125	2048×2048
	Canon CXDI	125	2048×2048
	Optima XR220	Unknown	2048×2048
Hospital-B	GE Discovery XR656	80-120	2048×2048
	GE Optima XR220	55	2048×2048
	DMS Platinum 43	80	2048×2048
	Canon CXDI	120	2048×2048

© 2023 Zhang Y et al. *JAMA Network Open.*

eTable 2. Classification performance of convolutional neural networks of symptomatic patients (*n*=5,996) in the retrospective test dataset using board reading as the reference

	Abnormal sign	AUC	AUPRC	Accuracy	Sensitivity	Specificity	F1-score
Lung parenchyma	Consolidation	0.90 (0.85-0.94)	0.55	0.94 (0.91-0.96)	0.62 (0.57-0.66)	0.97 (0.95-0.98)	0.76
	Small consolidation	0.76 (0.63-0.87)	0.44	0.83 (0.79-0.86)	0.60 (0.55-0.65)	0.84 (0.80-0.87)	0.70
	Patchy consolidation	0.90 (0.85-0.95)	0.48	0.95 (0.93-0.97)	0.55 (0.50-0.59)	0.98 (0.96-0.99)	0.69
	Nodule	0.70 (0.58-0.81)	0.39	0.93 (0.90-0.95)	0.33 (0.29-0.38)	0.96 (0.94-0.98)	0.49
	Calcification	0.72 (0.64-0.80)	0.29	0.86 (0.82-0.89)	0.45 (0.40-0.49)	0.90 (0.87-0.92)	0.59
	Mass	0.98 (0.97-0.99)	0.28	0.97 (0.96-0.99)	1.00 (0.99-1.00)	0.97 (0.96-0.99)	0.99
	Interstitial disease	0.95 (0.90-0.98)	0.31	0.99 (0.98-1.00)	0.33 (0.29-0.38)	1.00 (0.99-1.00)	0.50
	Cavity	0.92 (0.91-0.93)	0.37	0.93 (0.86-1.00)	0.68 (0.64-0.72)	0.93 (0.89-0.98)	0.79
	Hilar adenopathy	0.90 (0.80-0.98)	0.31	0.96 (0.94-0.98)	0.56 (0.52-0.61)	0.98 (0.96-0.99)	0.71
	Emphysema	0.97 (0.95-0.99)	0.35	0.97 (0.96-0.99)	0.83 (0.80-0.87)	0.98 (0.96-0.99)	0.90
	Pulmonary Edema	0.84 (0.79-0.88)	0.61	0.93 (0.88-0.99)	0.84 (0.76-0.92)	0.90 (0.89-0.91)	0.89
	Thickened bronchovascular markings	0.75 (0.64-0.83)	0.19	0.88 (0.85-0.91)	0.35 (0.31-0.40)	0.90 (0.87-0.93)	0.50
Mediastinum	Cardiomegaly	0.95 (0.93-0.97)	0.58	0.88 (0.85-0.91)	0.90 (0.86-0.92)	0.88 (0.84-0.91)	0.89
	Aortic unfolding	0.95 (0.92-0.98)	0.71	0.98 (0.68-0.99)	0.33 (0.29-0.38)	0.98 (0.96-0.99)	0.50
	Aortic arteriosclerosis	0.93 (0.91-0.95)	0.68	0.86 (0.82-0.89)	0.94 (0.91-0.96)	0.79 (0.75-0.83)	0.90
Pleura	Pneumothorax	0.96 (0.93-0.99)	0.42	0.97 (0.95-0.98)	0.86 (0.83-0.89)	0.98 (0.96-0.99)	0.91
	Pleural effusion	0.99 (0.98-0.99)	0.44	0.97 (0.95-0.98)	0.80 (0.76-0.84)	0.98 (0.97-0.99)	0.88
	Pleural thickening	0.79 (0.75-0.83)	0.38	0.77 (0.73-0.81)	0.69 (0.64-0.73)	0.79 (0.75-0.83)	0.73

© 2023 Zhang Y et al. *JAMA Network Open.*

	Pleural adhesion	0.94 (0.88-0.99)	0.51	0.99 (0.97-0.99)	0.50 (0.45-0.55)	0.99 (0.98-0.99)	0.66
	Pleural calcification	0.75 (0.65-0.86)	0.81	0.98 (0.97-0.99)	0.17 (0.14-0.21)	1.00 (0.99-1.00)	0.29
Thorax	Scoliosis	0.78 (0.74-0.82)	0.51	0.75 (0.71-0.79)	0.46 (0.41-0.51)	0.91 (0.87-0.93)	0.57
	PICC implant	0.63 (0.55-0.70)	0.47	0.75 (0.71-0.79)	0.33 (0.29-0.38)	0.79 (0.75-0.83)	0.46
	Pacemaker implant	1.00 (0.99-1.00)	0.49	1.00 (0.99-1.00)	1.00 (0.99-1.00)	1.00 (0.99-1.00)	1.00

AUC=area under the receiver operating characteristic curve; AUPRC=area under the precision-recall curve; PICC=peripherally inserted central catheter

© 2023 Zhang Y et al. *JAMA Network Open.*

eTable 3. Classification performance of convolutional neural networks of asymptomatic screening participants (*n*=2,130) in the retrospective test dataset using board reading as the reference

	Abnormal sign	AUC	AUPRC	Accuracy	Sensitivity	Specificity	F1-score
Lung parenchyma	Consolidation	0.88 (0.82-0.92)	0.37	0.98 (0.97-0.99)	0.41 (0.39-0.43)	0.99 (0.98-0.99)	0.57
	Small consolidation	0.84 (0.80-0.86)	0.29	0.96 (0.95-0.97)	0.22 (0.21-0.24)	0.99 (0.98-0.99)	0.36
	Patchy consolidation	0.90 (0.80-0.98)	0.35	0.99 (0.98-0.99)	0.33 (0.31-0.35)	0.99 (0.98-0.99)	0.50
	Nodule	0.80 (0.73-0.84)	0.41	0.98 (0.97-0.99)	0.38 (0.36-0.40)	0.98 (0.97-0.99)	0.54
	Calcification	0.90 (0.85-0.94)	0.26	0.98 (0.97-0.99)	0.46 (0.44-0.48)	0.99 (0.98-0.99)	0.63
	Interstitial disease	0.43 (0.42-0.44)	0.20	0.95 (0.94-0.97)	0.00 (0.00-0.01)	1.00 (0.99-1.00)	0.00
	Hilar adenopathy	0.64 (0.60-0.69)	0.41	0.82 (0.81-0.84)	0.70 (0.70-0.71)	1.00 (0.99-1.00)	0.76
	Emphysema	0.89 (0.88-0.90)	0.32	0.99 (0.99-1.00)	0.75 (0.73-0.77)	1.00 (0.99-1.00)	0.86
	Thickened bronchovascular markings	0.87 (0.77-0.94)	0.29	0.98 (0.98-0.99)	0.21 (0.19-0.23)	0.99 (0.99-1.00)	0.35
Mediastinum	Cardiomegaly	0.97 (0.95-0.99)	0.33	0.99 (0.98-0.99)	0.69 (0.67-0.71)	0.99 (0.98-0.99)	0.82
	Aortic unfolding	0.96 (0.95-0.98)	0.68	0.98 (0.98-0.99)	0.63 (0.62-0.64)	0.99 (0.98-0.99)	0.77
	Aortic arteriosclerosis	0.98 (0.98-0.99)	0.43	0.97 (0.96-0.98)	0.82 (0.80-0.84)	0.98 (0.97-0.99)	0.89
Pleura	Pleural thickening	0.94 (0.92-0.96)	0.46	0.99 (0.98-0.99)	0.62 (0.59-0.64)	0.99 (0.98-0.99)	0.76
	Pleural adhesion	0.75 (0.74-0.76)	0.37	0.99 (0.98-1.00)	0.25 (0.23-0.26)	1.00 (0.99-1.00)	0.40
	Pleural calcification	0.98 (0.96-1.00)	0.29	0.99 (0.99-1.00)	1.00 (0.99-1.00)	1.00 (0.99-1.00)	0.99
Thorax	Scoliosis	0.94 (0.91-0.96)	0.59	0.97 (0.96-0.98)	0.53 (0.50-0.55)	0.98 (0.98-0.99)	0.68

AUC=area under the receiver operating characteristic curve; AUPRC=area under the precision-recall curve

© 2023 Zhang Y et al. *JAMA Network Open.*

eTable 4. Disease prevalence in the prospective test dataset (*n*=5,091) based on board reading

		Symptomatic patients				Asymptomatic screening participants			
	Age group, *years*	Overall	18 to 44	45 to 59	60+	Overall	18 to 44	45 to 59	60+
	Subject, *n*	4175	1497	1039	1639	916	842	61	13
Lung parenchyma	Consolidation	67	15	23	29	NA	NA	NA	NA
	Small consolidation	537	117	190	232	2	0	1	1
	Patchy consolidation	374	64	138	176	4	0	1	3
	Nodule	235	46	62	127	9	1	4	4
	Calcification	35	3	2	30	1	0	0	1
	Mass	12	2	3	7	NA	NA	NA	NA
	Interstitial disease	10	0	2	8	NA	NA	NA	NA
	Cavity	NA	NA	NA	NA	NA	NA	NA	NA
	Hilar adenopathy	1	0	0	1	NA	NA	NA	NA
	Emphysema	12	1	1	10	1	0	0	1
	Pulmonary Edema	NA	NA	NA	NA	NA	NA	NA	NA
	Thickened bronchovascular markings	236	42	63	131	5	1	1	3
Mediastinum	Cardiomegaly	314	26	78	209	1	0	0	1
	Aortic unfolding	41	1	9	31	2	0	1	1
	Aortic arteriosclerosis	514	7	94	413	3	0	0	3
Pleura	Pneumothorax	62	14	22	26	NA	NA	NA	NA
	Pleural effusion	73	12	24	37	13	1	4	8
	Pleural thickening	204	42	75	87	140	29	51	60
	Pleural adhesion	54	14	17	23	5	1	1	4
	Pleural calcification	NA	NA	NA	NA	NA	NA	NA	NA
Thorax	Scoliosis	25	11	1	13	13	5	2	6
	PICC implant	903	124	269	510	NA	NA	NA	NA

© 2023 Zhang Y et al. *JAMA Network Open.*

	Pacemaker implant	26	0	6	20	NA	NA	NA	NA

NA=not available; PICC=peripherally inserted central catheter

© 2023 Zhang Y et al. *JAMA Network Open.*

eTable 5. Classification performance of convolutional neural networks of symptomatic patients (*n*=4,175) in the prospective test dataset using board reading as the reference

	Abnormal sign	AUC	AUPRC	Accuracy	Recall	Specificity	F1-score
Lung parenchyma	Consolidation	0.89 (0.86-0.92)	0.50	0.91 (0.88-0.94)	0.62 (0.60-0.65)	0.97 (0.94-1.00)	0.76
	Small consolidation	0.82 (0.60-1.00)	0.48	0.82 (0.60-1.00)	0.37 (0.27-0.47)	0.96 (0.80-1.00)	0.54
	Patchy consolidation	0.86 (0.70-1.00)	0.44	0.82 (0.66-0.97)	0.56 (0.45-0.66)	0.95 (0.87-1.00)	0.70
	Nodule	0.70 (0.62-0.79)	0.37	0.88 (0.78-0.98)	0.19 (0.16-0.21)	0.93 (0.84-1.00)	0.31
	Calcification	0.77 (0.75-0.78)	0.27	0.98 (0.97-1.00)	0.54 (0.53-0.55)	1.00 (0.99-1.00)	0.70
	Mass	0.85 (0.84-0.86)	0.24	0.99 (0.99-1.00)	0.59 (0.58-0.60)	1.00 (0.99-1.00)	0.74
	Interstitial disease	0.75 (0.74-0.76)	0.25	0.99 (0.99-1.00)	0.50 (0.49-0.51)	1.00 (0.99-1.00)	0.67
	Hilar adenopathy	0.99 (0.98-0.99)	0.33	0.99 (0.98-1.00)	0.62 (0.61-0.63)	1.00 (0.99-1.00)	0.77
	Emphysema	0.98 (0.97-0.99)	0.29	0.98 (0.97-0.99)	0.75 (0.74-0.76)	0.98 (0.97-0.99)	0.85
	Thickened bronchovascula r markings	0.68 (0.60-0.76)	0.31	0.53 (0.47-0.59)	0.72 (0.64-0.81)	0.52 (0.46-0.58)	0.60
Mediastinum	Cardiomegaly	0.89 (0.75-1.00)	0.60	0.92 (0.77-1.00)	0.38 (0.32-0.44)	0.96 (0.81-1.00)	0.55
	Aortic unfolding	0.85 (0.83-0.87)	0.12	0.98 (0.96-0.99)	0.22 (0.21-0.23)	0.98 (0.96-1.00)	0.36
	Aortic arteriosclerosis	0.85 (0.63-1.00)	0.54	0.87 (0.65-1.00)	0.34 (0.25-0.42)	0.95 (0.80-1.00)	0.50
Pleura	Pneumothorax	0.86 (0.62-1.00)	0.70	0.85 (0.77-1.00)	0.13 (0.09-0.16)	1.00 (0.99-1.00)	0.22
	Pleural effusion	0.89 (0.86-0.93)	0.69	0.78 (0.75-0.81)	0.12 (0.11-0.13)	0.98 (0.95-1.00)	0.21
	Pleural thickening	0.79 (0.71-0.87)	0.36	0.81 (0.72-0.88)	0.53 (0.48-0.58)	0.82 (0.74-0.90)	0.64
	Pleural adhesion	0.80 (0.78-0.82)	0.34	0.93 (0.90-0.95)	0.30 (0.28-0.32)	0.94 (0.91-0.96)	0.45
Thorax	Scoliosis	0.92 (0.91-0.93)	0.33	0.99 (0.98-1.00)	0.71 (0.69-0.72)	0.99 (0.98-1.00)	0.83
	PICC implant	0.69 (0.48-0.90)	0.20	0.77 (0.62-0.92)	0.61 (0.44-0.79)	0.98 (0.88-1.00)	0.75
	Pacemaker implant	0.99 (0.98-1.00)	0.88	0.99 (0.98-1.00)	0.62 (0.61-0.63)	0.99 (0.98-1.00)	0.76

Three abnormal signs were not detected, including cavitation, pulmonary edema, and pleural calcification. AUC=area under the receiver operating characteristic curve; AUPRC=area under the precision-recall curve; PICC=peripherally inserted central catheter

© 2023 Zhang Y et al. *JAMA Network Open.*

eTable 6. Classification performance of convolutional neural networks of asymptomatic screening participants (*n*=916) in the prospective test using board reading as the reference

	Abnormal sign	AUC	AUPRC	Accuracy	Recall	Specificity	F1-score
Lung parenchyma	Small consolidation	0.99 (0.98-0.99)	0.25	0.99 (0.980.99)	1.00 (0.99-1.00)	0.99 (0.98-0.99)	0.99
	Patchy consolidation	0.78 (0.77-0.79)	0.30	0.99 (0.98-0.99)	0.75 (0.74-0.76)	0.99 (0.98-0.99)	0.86
	Nodule	0.69 (0.68-0.70)	0.39	0.82 (0.81-0.83)	0.38 (0.37-0.39)	0.93 (0.92-0.94)	0.55
	Calcification	0.96 (0.95-0.97)	0.23	0.99 (0.98-0.99)	1.00 (0.98-1.00)	0.99 (0.98-0.99)	0.99
	Emphysema	1.00 (0.99-1.00)	0.19	0.99 (0.98-0.99)	1.00 (0.98-1.00)	0.99 (0.98-0.99)	0.99
	Thickened bronchovascula r markings	0.84 (0.83-0.85)	0.23	0.92 (0.91-0.93)	0.80 (0.79-0.81)	0.92 (0.91-0.93)	0.86
Mediastinum	Cardiomegaly	0.96 (0.95-0.97)	0.13	0.99 (0.98-0.99)	1.00 (0.99-1.00)	0.99 (0.99-1.00)	0.99
	Aortic unfolding	0.99 (0.98-0.99)	0.69	0.99 (0.98-0.99)	1.00 (0.99-1.00)	0.99 (0.99-1.00)	0.99
	Aortic arteriosclerosis	0.98 (0.98-0.99)	0.38	0.99 (0.98-0.99)	0.67 (0.66-0.68)	1.00 (0.99-1.00)	0.80
Pleura	Pleural effusion	0.98 (0.97-0.99)	0.45	0.99 (0.98-0.99)	1.00 (0.99-1.00)	1.00 (0.99-1.00)	1.00
	Pleural thickening	0.60 (0.56-0.65)	0.27	0.73 (0.68-0.78)	0.63 (0.59-0.67)	0.68 (0.63-0.73)	0.66
	Pleural adhesion	0.97 (0.96-0.98)	0.19	0.99 (0.98-0.99)	1.00 (0.99-1.00)	0.96 (0.95-0.97)	0.98
Thorax	Scoliosis	0.89 (0.88-0.90)	0.54	0.98 (0.97-0.99)	0.93 (0.92-0.94)	0.99 (0.99-1.00)	0.96

Ten abnormal signs were not detected, including consolidation, mass, interstitial disease, cavity, hilar adenopathy, pulmonary edema, pleural calcification, pneumothorax, PICC implant, and pacemaker implant. AUC=area under the receiver operating characteristic curve; AUPRC=area under the precision-recall curve

© 2023 Zhang Y et al. *JAMA Network Open.*

eTable 7. Multiple regression analysis on the significance of reporting time and BLEU score among three models

	Symptomatic patients		Asymptomatic screening participants	
	Coefficient	P value	Coefficient	P value
Reporting time				
Model	0.012	0.013	0.018	0.007
Age	0.005	0.033	0.006	0.300
Sex	0.020	0.014	0.011	0.083
Referral source	0.053	<0.001	NA	NA
Number of abnormal signs	0.008	0.048	0.083	<0.001
BLEU score				
Model	0.041	0.034	0.202	0.005
Age	0.001	0.923	0.006	0.243
Sex	0.010	0.083	-0.185	0.653
Referral source	0.013	0.027	NA	NA
Number of abnormal signs	-0.083	<0.001	-0.392	<0.001

The coding for categorical variables: Model (0: normal template, 1: NLP-generation model, 2: rule-based generation), Sex (0: female, 1: Male), Source of referral (0: emergency, 1: inpatients, 2: outpatients). BLEU = the bilingual evaluation understudy score; NA=not available.

© 2023 Zhang Y et al. *JAMA Network Open.*
