# Supplementary material for: Comparison of Chest Radiograph Captions Based on Natural Language Processing vs Completed by Radiologists
Source: JAMA Netw Open. 2023 Feb 8;6(2):e2255113. doi: 10.1001/jamanetworkopen.2022.55113 (PMC9909497; doi:10.1001/jamanetworkopen.2022.55113)
Supplement: Supplement 2. — Data Sharing Statement [file jamanetwopen-e2255113-s002.pdf]

Data Sharing Statement

Zhang. Comparison of Chest Radiograph Captions Based on Natural Language Processing vs Completed by Radiologists. *JAMA Netw Open*. Published February 08, 2023. doi:10.1001/jamanetworkopen.2022.55113

Data
Data available: No

Additional Information
Explanation for why data not available: The dataset is not publicly available to protect patient privacy, since the dataset contains patient-identifiable information. If any researcher wants to use the dataset for scientific purposes, please contact the corresponding author and apply for ethical approval from the data provider.
